# Supplementary material for: Identification and functional analysis of non-coding regulatory small RNA FenSr3 in Bacillus amyloliquefaciens LPB-18
Source: PeerJ. 2023 May 15;11:e15236. doi: 10.7717/peerj.15236 (PMC10194069; doi:10.7717/peerj.15236)
Supplement: Supplemental Information 4 [file peerj-11-15236-s004.zip › KO/CK-vs-T1_map/map05320.html]

KEGG PATHWAY: Autoimmune thyroid disease - Reference pathway


|  |  |
| --- | --- |
| **Autoimmune thyroid disease - Reference pathway** |  |

[
Pathway menu
| Organism menu
| Pathway entry
| Show description
| User data mapping
]

The classification of autoimmune throid disease (AITD) includes Hashimoto's thyroiditis (HT) or chronic autoimmune thyroiditis and its variants, Graves' disease (GD) and autoimmune atrophic thyroiditis or primary myxedema. HT is characterized by the presence of goitre, thyroid autoantibodies against thyroid peroxidase (TPO) and thyroglobulin (Tg) in serum and varying degrees of thyroid dysfunction. During HT, self-reactive CD4+ T lymphocytes (Th) recruit B cells and CD8+ T cells (CTL) into the thyroid. Disease progression leads to the death of thyroid cells and hypothyroidism. Both autoantibodies and thyroid-specific cytotoxic T lymphocytes (CTLs) have been proposed to be responsible for autoimmune thyrocyte depletion. In GD, the TSH-R is the most important autoantigen. Antibodies directed against it mimic the effects of the hormone on thyroid cells, TSH, stimulating autonomous production of thyroxine and triiodothyronine and causing hyperthyroidism. The presence of TSH-R-blocking antibodies that bind the TSH receptor in a similar fashion to the antibodies in patients with Grave's disease but that block rather than activate the receptor explains some cases of atrophic hypothyroidism.

|  |  |  |
| --- | --- | --- |
| Reference pathway | 184% 150% 122% 100% 82% 67% 55% | 图片下载 |
